# Genetic markers associated with bone strength and density in Rhode Island Red laying hens

**DOI:** 10.1016/j.psj.2025.105246

**Published:** 2025-05-02

**Authors:** Qiaoxian Yue, Martin Johnsson, Peter W Wilson, Björn Andersson, Matthias Schmutz, Cristina Benavides, Nazaret Dominguez-Gasca, Estefania Sanchez-Rodriguez, Alejandro B Rodriguez-Navarro, Ian C Dunn, Dirk-Jan de Koning

**Affiliations:** a Shanxi Agricultural University, Shanxi 030801, China; b Department of Animal Biosciences, Swedish University of Agricultural Sciences, Box 7023 750 07, Uppsala 756 51, Sweden; c The Roslin Institute and Royal (Dick) School of Veterinary Studies, University of Edinburgh, Midlothian, UK; d Lohmann Breeders, Cuxhaven 27472, Germany; e Departamento de Mineralogia y Petrologia, Universidad de Granada, Granada 18002, Spain

**Keywords:** Bone strength, Genome-wide association study, Laying hen, Rhode Island Red

## Abstract

Damage to the keel bone in commercial laying hens represent one of the greatest welfare issues in laying hens. This study aims to identify the DNA markers and candidate genes for bone strength and density traits in a Rhode Island Red laying hen population. We conducted genome-wide association studies (**GWAS**) on bone quality traits using a sample of 925 Rhode Island Red laying hens genotyped with a genotyping array consisting of 60 000 DNA markers. With a univariate linear mixed model, we identified 52 suggestive genetic markers located within 28 candidate genes that are associated with the humerus, keel, and tibia strength and density. We also found overlaps between the GWAS results for medullary bone score and tibia strength and density with published quantitative trait loci (**QTL**) for eggshell effective layer thickness and abdominal fat weight, respectively. Heritability estimates for the humerus stiffness, tibia stiffness, medullary bone score and minor bone diameter ranged from 0.21 to 0.34. Annotation term enrichment analysis of genes within 2 Megabases of suggestive markers found that mTOR signalling pathway, tryptophan metabolism, TGF-β signalling pathway, and apoptosis were significantly enriched. These loci do not overlap previously published associations, and thus appear to be novel.

## Introduction

High egg production makes laying hens prone to problems such as keel bone deformation and damage during the egg-laying period. Bone damage represents a considerable welfare and economic problems in commercial laying hens (Candelotto et al., 2017; Habig et al., 2021; Harlander-Matauschek et al*.,* 2015; Webster, 2004). As the age increases, the decrease of mineralized structural bone mass during the laying period leads to declining the health status and egg quality of laying hens. Currently, bone health is one of the main factors limiting the performance of extra-long laying hens (Alfonso-Carrillo et al*.,* 2021).

To date, a large number of QTLs and candidate genes that significantly affect chicken skeletal traits have been identified (Podisi et al*.,* 2012; Schreiweis et al*.,* 2005). A QTL region significantly associated with tibia and humerus breaking strength was identified on chromosome 1 in a hybrid F2 population of commercial purebred White Leghorn hen, which was recently fine-mapped and identified around the cystathionine β synthase (CBS) gene areas associated with osteoporosis (De Koning et al., 2020; Dunn et al., 2007). A GWAS analysis of hybrid F2 laying hens between a Chinese indigenous breed and a White Leghorn flock identified several candidate genes mapped to narrow regions related to bone development (Guo et al., 2017). GWAS combined selection signature analysis to analyze the genetic basis in an F2 population constructed by broiler and layer, and identified 21 candidate genes in 3 genomic regions significantly related to bone growth and development (Li et al., 2021).

The Rhode Island Red breed is one of the most common breeds in the world and is often used as a cross parent for many commercial layers (Kumar et al., 2002). Estimates of the heritability of bone quality traits in the Rhode Island Red population were recently reported, suggesting that bone quality could be alleviated through genetic selection (Dunn et al., 2021). The objective of this work was to identify associations and candidate genes for bone strength and density traits in the Rhode Island Red population, including the tibia breaking strength, tibia density, tibia stiffness, cortical thickness, the major and minor diameter of tibia, the humerus breaking strength, humerus density, humerus stiffness, medullary bone score, and the keel bone density using Illumina 60 K SNP array data.

## Materials and methods

### Animals and phenotypes

We used data from 925 Rhode Island Red hens previously studied by (Dunn et al., 2021; Sallam et al., 2023). Hens were housed with a companion hen in cages that were equipped with a perch. All hens had free access to feed and water and were managed in the same environment. Samples were collected at 68 weeks of age from four hatches as previously described. The breaking strength of the humerus and tibia was evaluated by a three-point bending test, and the density test of the humerus, tibia and keel was collected by an X-ray scanner as the previously described (Dunn et al., 2021). Descriptive statistics for body weight and bone traits are listed in Table 1. Covariates for genome-wide association studies were identified by fitting body weight, housing system, and hatch of week in a multiple regression model with bone traits as response variable, and birds that had high leverages (greater than three times the standard deviation) were removed.

**Table 1:** Descriptive statistics for bone traits.

Traits/unit	N	Min	Max	Mean	SD	CV/%	Number of SNPs
Thickness/mm	902	0.254	1.144	0.598	0.107	17.934	30,753
Tibia density/mm_Al_equiv	892	1.687	3.089	2.357	0.241	10.237	30,750
Tibia breaking strain/N	889	98.700	387.000	226.600	50.083	22.103	30,746
Tibia stiffness/N_mE	881	14.803	57.091	33.912	7.341	21.645	30,743
Minor diameter/mm	883	5.857	8.160	6.993	0.437	6.246	30,754
Major diameter/mm	884	6.953	10.023	8.527	0.576	6.750	30,740
Keel density/mm_Al_equiv	866	0.573	1.088	0.825	0.097	11.740	30,749
Humerus density/mm_Al_equiv	825	0.947	2.361	1.432	0.304	21.255	30,756
Humerus breaking strain/N	861	74.400	292.100	157.500	45.539	28.911	30,406
Humerus stiffness/N_mE	862	8.822	42.069	23.461	6.091	25.963	30,744
Medullary bone score	879	-	-	-	-	-	30,759

### Genotypes

The hens were genotyped with the Illumina 60 K chicken SNP array which contained 57,636 SNPs across 33 autosomes and two sex chromosomes (Groenen et al., 2011). We first discarded SNPs with unknown physical position and repeated genomic coordinates. The PLINK software (http://pngu.mgh.harvard.edu/purcell/plink/; Purcell et al., 2007) was then used to control the quality of individuals by removing those with a missing genotype frequency higher than 0.05 and omitting SNPs with a minor allele frequency lower than 0.01 or a Hardy-Weinberg equilibrium *P* value lower than 1 × 10^-4^. The number of variants located on autosomes eligible for the following analysis is shown in Table 1.

### Genome-wide association analysis

Association between each genetic locus and bone quality trait, and heritability of each trait were evaluated under a univariate linear mixed model in the GEMMA (Zhou and Stephens, 2012) program with the command “-lmm 1″. The statistical model is shown as follows:

*y* = **W***α* + **X***β* + ***u****+ ε*; **u**∼MV*N_n_* (0, λ*τ*^−1^**K**); **ε**∼MNVn (0, τ^−1^**I**_n_)

Where y is a vector of phenotypic values. **W** is a matrix of covariates (hatch, house and body weight) including a column of 1 s, α is a vector of the corresponding coefficients including the intercept. **X** is a vector of locus genotypes, β is the effect size of the locus. **u** is a vector of additive genetic effects, λ is the ratio of the random effect variance to the residual error variance, *τ*^−1^ represents the variance of the residual errors and **K** is a kinship matrix calculated from a pruned set of total SNP loci obtained by the command “-gk 2″ in GEMMA. **ε** is a vector of residual errors, **I***_n_* is an identity matrix.

Body weight, hatch and house were included as fixed effects in the genome-wide association study because of significant associations with traits when analyzed in a linear model (Supplementary Table 1).

In this study, a 1.6 × 10^−6^ *P* value was used as the genome-wide significance level using Bonferroni correction (0.05/31000) and the suggestive significance threshold for *P*-value was arbitrarily set to 1 × 10^−4^ to provide a less conservative presentation of the results. The “QQman” package in the R software (Turner, 2018) was used to draw Manhattan and quantile—quantile plots.

### SNP identification, candidate gene annotation and QTL detection

The GWAS peak SNPs were physically localized on the Galgal 6.0 reference genome assembly by the Ensembl Genes database (https://www.ensembl.org/index.html) and candidate genes closest to each tag SNP were identified. Based on the GWAS results, we used the location of the significant SNPs to search for candidate genes with GALLO R package (Fonseca et al., 2020) by entering the position of a SNP and ±500 kb. Furthermore, the chicken QTLdb (https://www.animalgenome.org/cgi-bin/QTLdb/GG/index) (Hu et al., 2013) was used to find previously detected associations perform QTL enrichment testing with *P*_adj_<0.05 and N_QTLS>2.

To identify a large set of suggestive SNPs for annotation term enrichment, we used a threshold to 0.0004, and a 2-Mb region was defined around each suggestive SNP. To investigate the biological associations among the candidate genes within these regions of all the GWAS lead SNPs, we performed GO and KEGG enrichment analysis by the DAVID database (https://david.ncifcrf.gov) (Huang da et al., 2009; Sherman et al., 2022).

### Power analysis

To explore the power characteristics of the study, we used the genpwr R package (Moore et al., 2019), assuming a linear model, additive genetics and a significance threshold like in this study. We estimated the power to detect loci explaining 0.1 %, 1 %, 5 %, 10 % of the variance at different sample sizes, as well as the effect size that could be detected with 80 % power assuming a sample size of 850 individuals. The results are shown in Supplementary Fig. 1.

## Results

### Genome-wide association studies

**Fig. 1:**
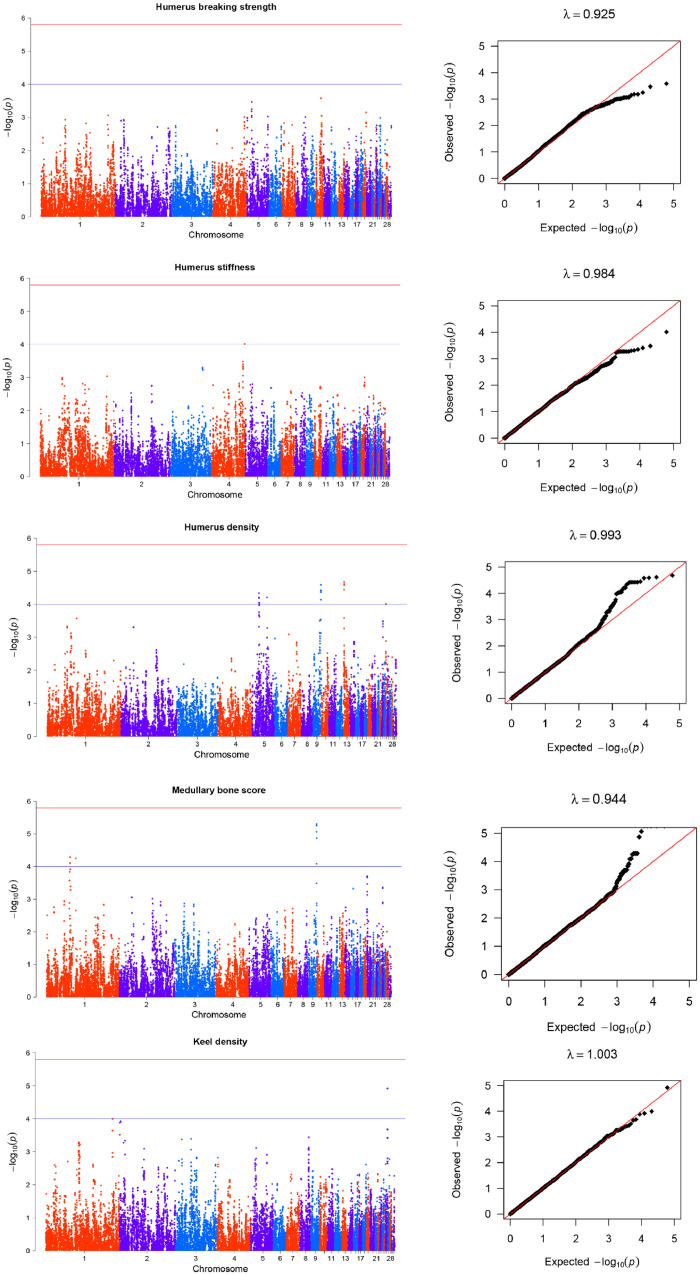
Manhattan and QQ plots for the association analyses of humerus and keel traits. In the Manhattan plots, -log10(P-value) of the filtered high-quality SNPs (y-axis) is plotted against their genomic positions (x-axis). In the Q-Q plots, -log10(p) of observed association statistics on the Y-axis were compared to those of the association statistics expected under the hypothesis of no association on the X-axis. The solid line represents concordance between observed and expected values. Genomic inflation factor, λ, is shown for each dataset.

Thirty-eight SNPs were associated with humerus traits. The Q-Q plots were used to illustrate the level of potential P-value inflation. The average genomic inflation factor (λ) is 0.962 (Fig. 1, Table 2), which indicated the absence of any obvious population stratification and the reliability of GWAS results. For keel density and humerus stiffness, there was only one suggestive SNP associated, with GGA 26 and 4, respectively. Fourteen significant SNPs associated with medullary bone score were clustered in two chromosomal regions, on GGA1 and 9. A total of twenty-two suggestive loci distributed on GGA 5, 9, 13, and 25 were associated with humerus density. Furthermore, eight suggestive SNPs associated with the medullary bone score were also found to be associated with humerus density at a suggestive level.

**Table 2:** Description of SNPs significantly associated with bone quality traits in Rhode Island Red hens.

Trait	Peak SNP	CHR	Pos (bp)	A1	A0	MAF	Beta (s.e)	Var (%)	P-value	Nearest gene or transcript
Humerus density	Gga_rs15674900	5	17,748,696	T	C	0.341	−0.076	0.0189	6.105E-05	CCND1
	Gga_rs14519806	5	17,756,573	A	G	0.341	−0.076	0.0189	6.449E-05	CCND1
	GGaluGA277535	5	17,793,200	A	G	0.342	−0.077	0.0189	4.601E-05	CCND1
	Gga_rs15675036	5	17,816,747	A	G	0.343	−0.075	0.0189	9.051E-05	FGF19
	GGaluGA277719	5	18,592,354	T	C	0.323	−0.075	0.0190	9.180E-05	ENSGALG00000049369
	Gga_rs14957234	5	18,611,927	T	C	0.323	−0.075	0.0190	9.047E-05	ENSGALG00000049369
	GGaluGA277835	5	18,967,730	A	G	0.317	−0.074	0.0190	9.270E-05	ELF5
	GGaluGA284473	5	39,327,053	T	C	0.255	−0.085	0.0211	6.100E-05	TMEM63C
	Gga_rs15987089	9	22,571,590	A	G	0.408	0.073	0.0171	4.978E-06	MFSD1
	Gga_rs16680382	9	22,641,697	T	C	0.382	0.072	0.0173	5.536E-06	RSRC1
	GGaluGA343964	9	22,694,487	A	G	0.381	0.072	0.0174	8.577E-06	RSRC1
	Gga_rs14682747	9	22,716,989	T	C	0.382	0.072	0.0173	5.536E-06	RSRC1
	GGaluGA343975	9	22,718,522	A	G	0.382	0.072	0.0173	5.536E-06	RSRC1
	Gga_rs13736850	9	22,749,245	T	G	0.382	0.072	0.0173	5.536E-06	RSRC1
	Gga_rs14682782	9	22,755,016	A	G	0.382	0.072	0.0173	5.536E-06	RSRC1
	GGaluGA344028	9	22,808,170	A	G	0.383	0.069	0.0173	1.351E-05	SHOX2
	Gga_rs14057603	13	2,613,738	A	G	0.378	0.073	0.0175	3.565E-05	PCDHGC3
	GGaluGA000809	13	2,617,085	A	G	0.390	0.073	0.0171	2.099E-05	PCDHGC3
	GGaluGA090604	13	2,723,555	T	C	0.382	0.074	0.0175	2.641E-05	ENSGALG00000034005
	Gga_rs14057466	13	2,786,047	A	G	0.390	0.073	0.0172	2.445E-05	PCDH1
	GGaluGA194683	25	363,950	T	C	0.244	0.075	0.0192	9.800E-05	POGZ
	GGaluGA194718	25	421,590	A	G	0.244	0.075	0.0192	9.800E-05	SNX27
Humerus stiffness	GGaluGA270428	4	88,558,830	A	G	0.323	−12535.990	3200.4710	9.684E-05	PTPRA
Keel density	Gga_rs16204910	26	5,100,286	T	C	0.360	−0.033	0.0075	1.207E-05	ENSGALG00000031807
Medullary bone score	Gga_rs13878250	1	63,701,762	A	G	0.178	0.370	0.0934	7.918E-05	RERGL
	GGaluGA022457	1	63,723,936	A	G	0.180	0.379	0.0931	5.159E-05	RERGL
	Gga_rs13878420	1	63,853,470	T	C	0.180	0.379	0.0931	5.159E-05	RERGL
	GGaluGA022507	1	63,896,924	A	G	0.180	0.379	0.0931	5.159E-05	RERGL
	GGaluGA026797	1	79,347,934	A	G	0.372	−0.295	0.0728	5.604E-05	HAO2
	GGaluGA343801	9	22,427,285	A	G	0.340	0.285	0.0720	8.173E-05	ENSGALG00000039468
	Gga_rs15987089	9	22,571,590	A	G	0.412	0.322	0.0700	4.978E-06	MFSD1
	Gga_rs16680382	9	22,641,697	A	G	0.386	0.324	0.0709	5.536E-06	RSRC1
	GGaluGA343964	9	22,694,487	A	G	0.385	0.318	0.0711	8.577E-06	RSRC1
	Gga_rs14682747	9	22,716,989	A	G	0.386	0.324	0.0709	5.536E-06	RSRC1
	GGaluGA343975	9	22,718,522	A	G	0.386	0.324	0.0709	5.536E-06	RSRC1
	Gga_rs13736850	9	22,749,245	A	G	0.386	0.324	0.0709	5.536E-06	RSRC1
	Gga_rs14682782	9	22,755,016	A	G	0.386	0.324	0.0709	5.536E-06	RSRC1
	GGaluGA344028	9	22,808,170	A	G	0.386	0.310	0.0709	1.351E-05	SHOX2
Tibia breaking strength	Gga_rs14145402	2	17,645,348	T	C	0.483	−11.001	2.5930	2.441E-05	PIP4K2A
	Gga_rs15032791	17	3,353,238	A	G	0.097	−17.884	4.5094	7.903E-05	PAPPA
Tibia density	Gga_rs14517040	5	13,545,074	A	G	0.444	−0.052	0.0124	3.138E-05	KCNQ1
	Gga_rs16468468	5	13,606,911	A	G	0.445	−0.052	0.0124	3.247E-05	KCNQ1
Tibia stiffness	Gga_rs14148617	2	20,486,067	T	G	0.463	15535.890	3969.8630	9.805E-05	FAM171A1
	Gga_rs14150107	2	21,774,232	T	C	0.480	−15952.890	4046.8040	8.723E-05	CDK14
	Gga_rs14150254	2	21,912,734	A	G	0.480	−16256.690	4038.7280	6.186E-05	CDK14
	Gga_rs14620039	7	26,263,298	T	C	0.215	−18345.500	4671.5250	9.275E-05	ENSGALG00000026460
	Gga_rs14620154	7	26,367,786	A	G	0.229	−18468.990	4537.1030	5.113E-05	SLC15A2
	Gga_rs14620196	7	26,384,936	T	C	0.216	−18476.380	4676.4090	8.411E-05	SLC15A2
	Gga_rs14620221	7	26,411,766	T	C	0.230	−19411.710	4536.9180	2.088E-05	SLC15A2
	Gga_rs15008991	14	7,144,668	T	C	0.021	54588.740	13897.9700	9.247E-05	CACNG3
	Gga_rs15009148	14	7,330,887	T	C	0.026	48740.250	12382.3700	8.934E-05	ENSGALG00000006140
Bone diameter major	Gga_rs16470339	5	15,066,389	A	C	0.215	0.141	0.0357	7.961E-05	MUC5B

**Fig. 2:**
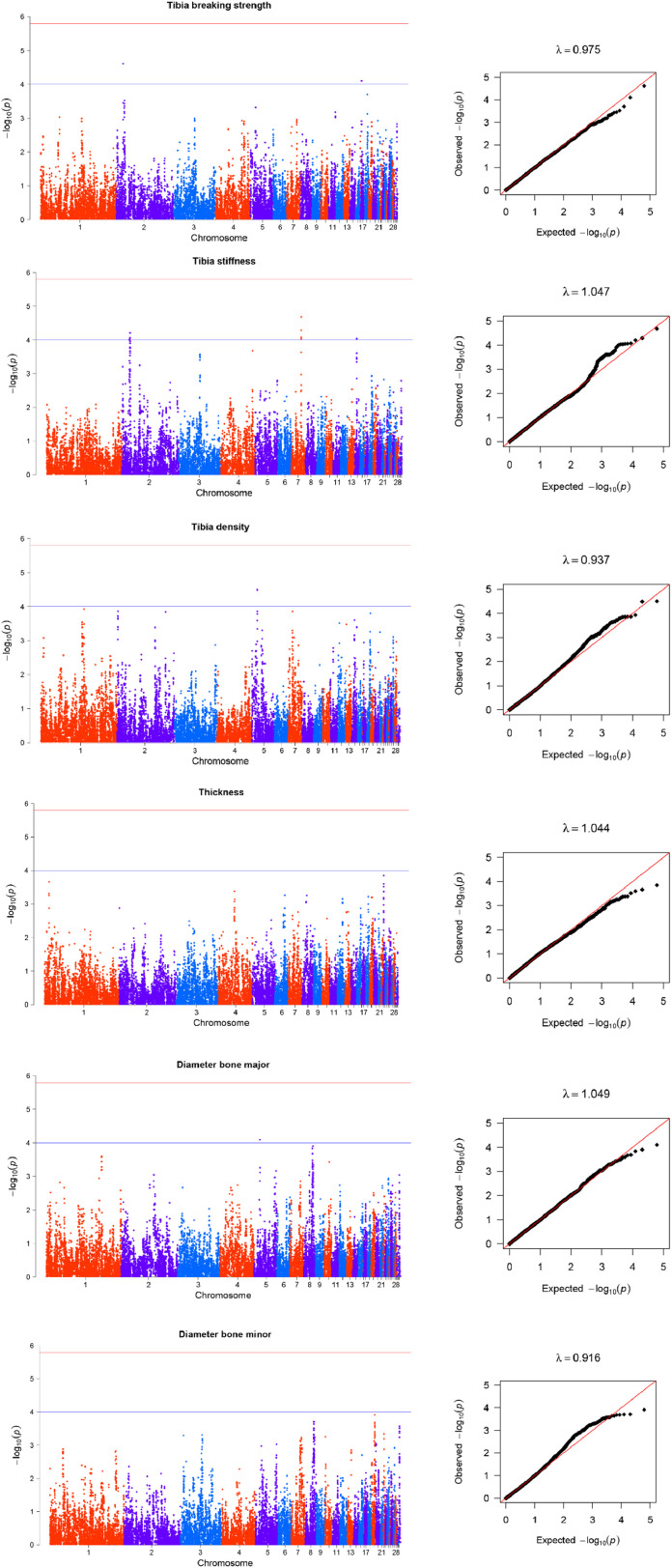
Manhattan and QQ plots for the association analyses of tibia traits. In the Manhattan plots, -log10(P-value) of the filtered high-quality SNPs (y-axis) is plotted against their genomic positions (x-axis). In the Q-Q plots, -log10(p) of observed association statistics on the Y-axis were compared to those of the association statistics expected under the hypothesis of no association on the X-axis. The solid line represents concordance between observed and expected values. Genomic inflation factor, λ, is shown for each dataset.

Fourteen SNPs were associated with tibia strength and density traits on GGA 2, 5, 7, 14, and 17, and the average genomic inflation factor (λ) is 1.000 (Fig. 2, Table 2). Two suggestive SNPs for tibia density were located on GGA 5, respectively. Two suggestive SNPs for tibia breaking strength, on GGA 2 and 17, respectively. And only one suggestive SNP for major bone diameter was located on GGA 5. A total of nine suggestive SNPs distributed on GGA 2, 7and 14 were associated with tibia stiffness. No SNP was associated with cortical bone thickness and minor bone diameter at the GWAS significance level.

### QTL and annotation term enrichment

**Fig. 3:**
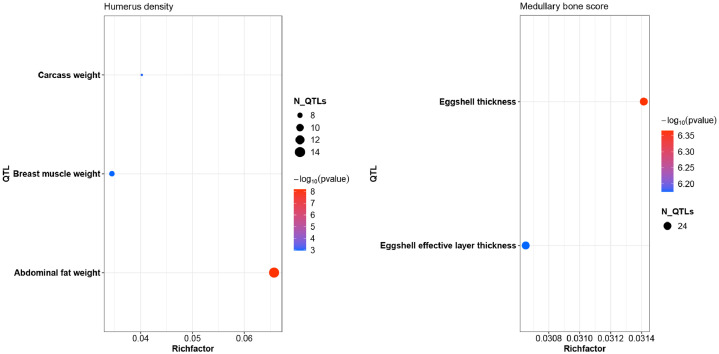
Enrichment of QTL overlapping humerus traits quality associations.

**Fig. 4:**
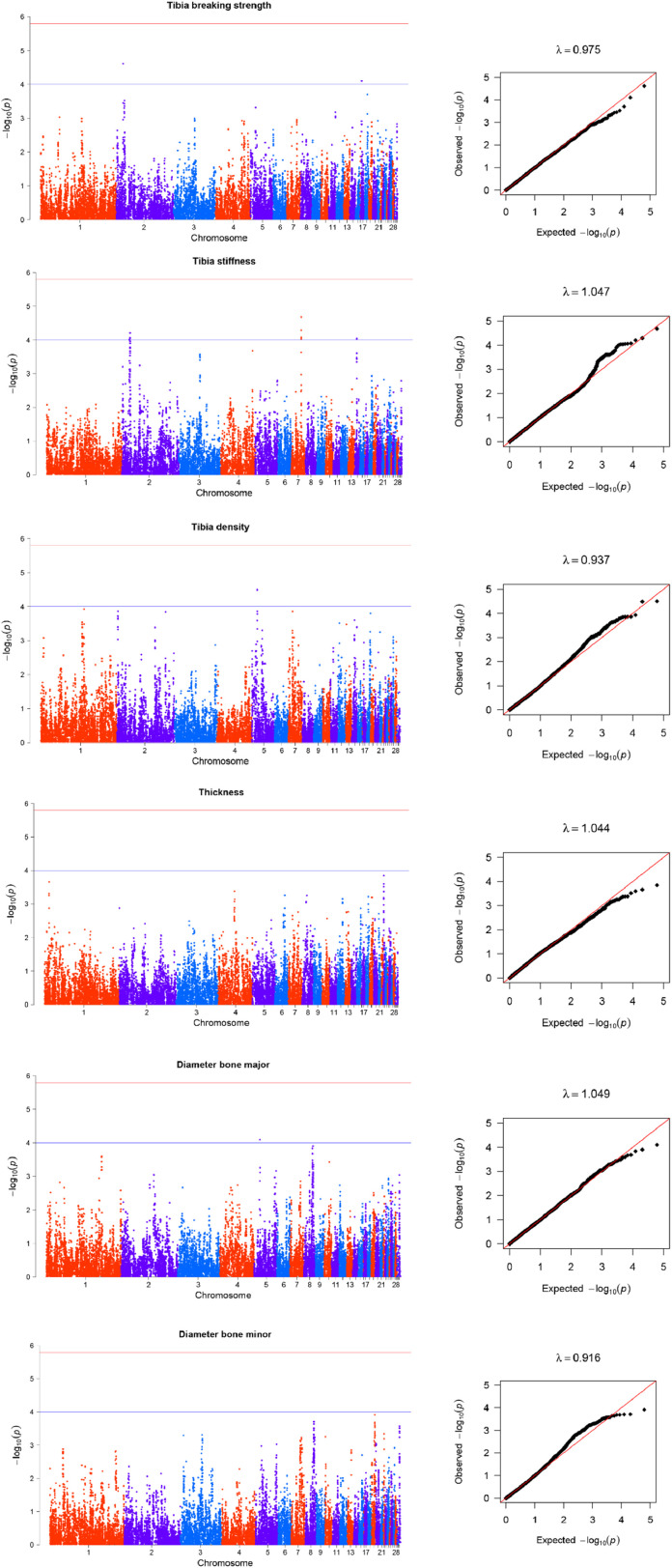
Enrichment of QTL overlapping tibia traits quality associations.

We overlapped the regions from genome-wide association studies with published quantitative trait loci from ChickenQTLdb. The results showed that the abdominal fat weight QTL overlapped regions for five bone traits, while eggshell thickness QTL overlapped the medullary bone score regions. There were also overlaps with breast, carcass and body weight QTL (Fig. 3, Fig. 4).

Annotation term enrichment analysis revealed 12 Gene Ontology terms with FDR<0.1, among them, keratin filament for the minor bone diameter, and adenylate cyclase-modulating G-protein coupled receptor signalling pathway for the tibia density. Seven terms were enriched for the medullary bone score including K‑threo-aldose 1-dehydrogenase activity, bile acid transmembrane transporter activity, G-protein coupled purinergic nucleotide receptor activity, and humerus density, three terms for tibia breaking strength which are ATPase activity, coupled to transmembrane movement of substances and voltage-gated calcium channel activity (Table 3). According to the KEGG database, eight pathway annotations were significantly enriched, including mTOR signalling pathway, tryptophan metabolism, TGF-β signalling pathway, and apoptosis (Table 4).

**Table 3:** GO term enrichment test involving the genes within 2 Mb of the SNPs with *P* < 0.0004.

Trait	ID	Term	Gene count	*P*-Value	FDR
Bone diameter minor	GO:0045095	Keratin filament	11	1.88E-15	3.48E-13
Medullary bone score	GO:0047834	D‑threo-aldose 1-dehydrogenase activity	5	1.98E-05	4.96E-03
Medullary bone score	GO:0015125	Bile acid transmembrane transporter activity	4	3.94E-05	4.96E-03
Medullary bone score	GO:0045028	G-protein coupled purinergic nucleotide receptor activity	4	6.25E-05	5.25E-03
Medullary bone score	GO:0004032	alditol:NADP+ 1-oxidoreductase activity	4	1.32E-04	8.31E-03
Medullary bone score	GO:0015347	Sodium-independent organic anion transmembrane transporter activity	4	1.80E-04	9.07E-03
Tibia breaking strength	GO:0042626	ATPase activity, coupled to transmembrane movement of substances	5	1.41E-04	2.18E-02
Tibia breaking strength	GO:0005245	Voltage-gated calcium channel activity	4	6.11E-04	4.74E-02
Medullary bone score	GO:0015721	Bile acid and bile salt transport	4	1.74E-04	6.60E-02
Medullary bone score	GO:0043252	Sodium-independent organic anion transport	4	2.38E-04	6.60E-02
Tibia breaking strength	GO:0005886	Plasma membrane	26	4.81E-04	6.64E-02
Tibia density	GO:0007188	Adenylate cyclase-modulating G-protein coupled receptor signaling pathway	9	4.71E-05	7.31E-02

**Table 4:** Gene set enrichment test involving the genes within 2 Mb of the SNPs with *P* < 0.0004.

Term	Gene count	*P*-value
mTOR signaling pathway	29	5.813E-03
Influenza A	26	1.487E-02
ABC transporters	11	1.992E-02
Apoptosis	24	2.027E-02
Tryptophan metabolism	10	2.704E-02
Fanconi anemia pathway	12	2.721E-02
Folate biosynthesis	8	3.541E-02
TGF-beta signaling pathway	18	4.755E-02

## Discussion

This study aimed to identify potential candidate genes associated with genetic variation in humerus and tibia bone strength and density using blood samples from 925 Rhode Island Red hens. There was a lack of major large-effect loci, but we identified suggestive genetic associations for humerus (38 SNPs) and tibia (14 SNPs) traits. These loci did not overlap previously published bone QTL, and thus appear to be novel (Johnsson et al., 2022; Raymond et al., 2018). There was also no overlap between suggestive associations for bone composition traits identified in the same population (Sallam et al., 2023).

The relative absence of major loci in the presence of substantial genomic heritability reaffirms the polygenicity of bone strength and density traits and suggests that genomic prediction of bone traits may be fruitful. Indeed, the potential for genomic prediction of tibial bone strength within the Rhode Island Red pure line has been demonstrated (Sallam et al., 2025).

### Genetic association with the humerus traits

In the current study, the top SNP is located within the *MFSD1* (major facilitator superfamily domain containing 1) gene on GGA9 and is the signal most strongly associated with humerus density and medullary bone score, referred to as pleiotropy, which is prevalent in the genetic architecture of chickens. A significant phenotypic correlation existed between humerus density and medullary bone score (*P* < 0.01). MFSD1 is an unglycosylated protein, and the immunofluorescence results showed that MFSD1 is present in the lysosomes of differentiated osteoclasts (Massa Lopez et al., 2016). The trabecular bone of vertebrae in MFSD1-deficient mice had decreased BMD and BV/TV ratio, increased osteoblast number and osteoclast activity, leading to the decline of bone mineral density (Lopez, 2018). Four medullary bone score associations on GGA1 are located within 166 kb upstream of the *RERGL* (Ras-like estrogen-regulated growth inhibitor-like) gene and overlap with QTLs for eggshell effective layer thickness and eggshell thickness in crossed F2 individuals (Duan et al., 2016; Liu et al., 2011). Mineralized medullary bone serves as a “calcium store” for eggshell formation, and the medullary bone undergoes turnover during a daily egg-laying cycle (Mueller et al., 1969).

Some markers that were found to be associated with humerus density on GGA5 overlap with the QTL for carcass weight in crosses between New Hampshire and White Leghorn chicken (Nassar et al., 2012), and QTL for breast muscle weight and abdominal fat weight that were detected in White Plymouth Rock (Atzmon et al., 2008). Among the markers, three suggestive markers are located within *CCND1* (Cyclin D1) gene, Let-7b targets *CCND1* to regulate osteoblast differentiation in mouse MC3T3-E1 cells (Wang and Cai, 2020). MiR-23b-3p functioned as a positive factor through regulating cell cycle, proliferation, apoptosis, and differentiation of MC3T3-E1 cells via targeting *CCND1* (Wang and Zhao, 2021). A suggestive marker overlaps with the *FGF19* (Fibroblast growth factor 19) gene on GGA5, which may inhibit osteoclastogenesis by regulating osteoprotegerin (OPG)/NF-κb ligand (RANKL) axis receptor activator (Guo et al., 2022) and enhance osteogenic differentiation via the Wnt/β-linked protein pathway that is associated with the regulation of osteogenic differentiation and bone formation (Teufel and Hartmann, 2019).

### Genetic association with the tibia traits

The marker on GGA2 associated with tibia breaking strength located within the *PIP4K2A* (phosphatidylinositol-5-phosphate 4-kinase type 2 alpha) gene, is actively involved in regulating intracellular cholesterol transport and negatively correlated with bone mineral density, mutations in this gene did not affect humerus mineral density of laying hens (Hu et al., 2018a). Two tibia stiffness associations on GGA2 (bp: 21,774,232 and 21,912,734) are located downstream the *CDK14* (Cyclin dependent kinase 14) gene, a novel cell cycle protein-dependent kinase, is a cell cycle regulator whose upregulation indicates increased cell proliferation during peri‑implant bone healing (Davidson and Niehrs, 2010). *SLC15A* (Solute carrier family 15 member 2) associated with tibia stiffness located on GGA7 is a transmembrane transporter protein expressed in cell membranes and organelle membranes. Osteoclasts can be formed from tissue-specific macrophages in inflammatory and immunological environments (Sun et al., 2021). In mice, *SLC15A* is highly expressed in mature immune cells and macrophages in the bone marrow (Hu et al., 2018b). A marker association with the tibia density on GGA5 (bp: 13,606,911) is located within the *KCNQ1* (Potassium voltage-gated channel subfamily Q member 1) gene. lnc-KCNQ1OT1 (KCNQ1 opposite strand/ antisense transcript 1), often regarded as an imprinted lincRNA, regulates osteogenic differentiation of mesenchymal stem cells by miR-214/BMP2 axis (Wang et al., 2019) and alleviates osteoclast differentiation (Zhang et al., 2021).

These markers that were found to be associated with tibia strength and density traits on GGA 2, GGA5, and GGA7 all overlap with a QTL for abdominal fat weight in a chicken interline cross with White Plymouth Rock background (Atzmon et al., 2008). A study on Korean men showed that abdominal obesity is a risk factor for osteoporosis whether in young or old men (Kim et al., 2019), the research on Chinese men showed that abdominal fat and visceral fat mass have negative effects on bone microstructure (Lv et al., 2016). On the contrary, excess fat increases the mechanical load on bones and results in higher bone mineral density (Rosen and Bouxsein, 2006). Therefore, the potential mechanism between abdominal fat and bone mass in laying hens deserves further investigation.

### The annotation term enrichment for each bone trait

The candidate genes detected in this study are mainly related to skeletal development, mTORC1 and mTORC2 both implicated in regulating osteoblast differentiation and function (Chen and Long, 2015). Besides, mTORC1 and mTORC2 signaling play an important and minor role in endochondral skeletal development, respectively. TGF-α signaling pathway have diverse functions in osteoblast differentiation, skeletal development, growth plate development, and bone formation (Chen et al., 2012; Wu et al., 2016). The primary active metabolites of tryptophan metabolism are serotonin, melatonin, and kynurenine, all of which play vital roles in bone biology (Al Saedi et al., 2020). Serotonin is divided into brain- and gut-derived, and both are functionally independent. Among them, brain-derived serotonin enhances bone formation and inhibits bone resorption (Ducy and Karsenty;, 2010). Gut-derived serotonin inhibits bone formation with no changes in bone resorption (de Vernejoul et al., 2012; Yadav et al., 2008). Increasing kynurenine levels results in accelerated skeletal aging by impairing osteoblastic differentiation and increasing osteoclastic resorption (Refaey et al., 2017).

## Conclusion

In the current study, we conducted an association analysis based on the SNPs data and humerus, keel and tibia traits of the Rhode Island Red hens. We obtained 52 suggestive SNPs loci, and there was a total of 28 genes near these SNPs. These loci do not overlap previously published associations, and thus appear to be novel.

**Supplementary Fig. 1.** Power analysis for a GWAS design. The vertical dashed line indicates the size of the present study while the horizontal line corresponds to a statistical power of 80 %

## Declaration of competing interest

The authors declare the following financial interests/personal relationships which may be considered as potential competing interests: Two of the co-authors are employes by Lohmann Animal Breeding: Matthias Schmutz and Björn Andersson. Lohmann Animal Breeding provided the birds for this study but did not perform the analysis. All results are presented in full without any embargo or restrictions by Lohmann Animal Breeding. If there are other authors, they declare that they have no known competing financial interests or personal relationships that could have appeared to influence the work reported in this paper.

## Appendix Supplementary materials

Image, application 1

## References

[bib0001] Al Saedi A., Sharma S., Summers M.A., Nurgali K., Duque G. (2020). The multiple faces of tryptophan in bone biology. Exp. Gerontol..

[bib0002] Alfonso-Carrillo C., Benavides-Reyes C., de Los Mozos J., Dominguez-Gasca N., Sanchez-Rodríguez E., Garcia-Ruiz A.I., Rodriguez-Navarro A.B. (2021). Relationship between bone quality, egg production and eggshell quality in laying hens at the end of an extended production cycle (105 weeks). Animals.

[bib0003] Atzmon G., Blum S., Feldman M., Cahaner A., Lavi U., Hillel J. (2008). QTLs detected in a multigenerational resource chicken population. J. Hered..

[bib0004] Candelotto L., Stratmann A., Gebhardt-Henrich S.G., Rufener C., van de Braak T., Toscano M.J. (2017). Susceptibility to keel bone fractures in laying hens and the role of genetic variation. Poult. Sci..

[bib0005] Chen G., Deng C., Li Y.P. (2012). TGF-β and BMP signaling in osteoblast differentiation and bone formation. Int. J. Biol. Sci..

[bib0006] Chen J., Long F. (2015). mTORC1 signaling promotes osteoblast differentiation from preosteoblasts. PLoS One.

[bib0007] Davidson G., Niehrs C. (2010). Emerging links between CDK cell cycle regulators and wnt signaling. Trends Cell Biol..

[bib0008] De Koning D.J., Dominguez-Gasca N., Fleming R.H., Gill A., Kurian D., Law A., McCormack H.A., Morrice D., Sanchez-Rodriguez E., Rodriguez-Navarro A.B., Preisinger R., Schmutz M., Šmídová V., Turner F., Wilson P.W., Zhou R., Dunn I.C. (2020). An eQTL in the cystathionine beta synthase gene is linked to osteoporosis in laying hens. Genet. Sel. Evol..

[bib0009] De Vernejoul, M.C., Collet, C., and Chabbi-Achengli Y., 2012. Serotonin: good or bad for bone. Bonekey Rep. 1:120. doi:10.1038/bonekey.2012.120. 10.1038/bonekey.2012.120 PMC3727814 23951501

[bib0010] Duan Z., Sun C., Shen M., Wang K., Yang N., Zheng J., Xu G. (2016). Genetic architecture dissection by genome-wide association analysis reveals avian eggshell ultrastructure traits. Sci. Rep..

[bib0011] Ducy P., Karsenty G. (2010). The two faces of serotonin in bone biology. J. Cell Biol..

[bib0012] Dunn I.C., De Koning D.J., McCormack H.A., Fleming R.H., Wilson P.W., Andersson B., Schmutz M., Benavides C., Dominguez-Gasca N., Sanchez-Rodriguez E., Rodriguez-Navarro A.B. (2021). No evidence that selection for egg production persistency causes loss of bone quality in laying hens. Genet. Sel. Evol..

[bib0013] Dunn I.C., Fleming R.H., McCormack H.A., Morrice D., Burt D.W., Preisinger R., Whitehead C.C. (2007). A QTL for osteoporosis detected in an F2 population derived from White Leghorn chicken lines divergently selected for bone index. Anim. Genet..

[bib0014] Fonseca P.A.S., Suárez-Vega A., Marras G., Cánovas Á. (2020). GALLO: an R package for genomic annotation and integration of multiple data sources in livestock for positional candidate loci. Gigascience.

[bib0015] Groenen M.A., Megens H.J., Zare Y. (2011). The development and characterization of a 60K SNP chip for chicken. BMC Genom..

[bib0016] Guo A., Li K., Tian H.C., Tao B.L., Xiao Q., Jiang D.M. (2022). FGF19 protects against obesity-induced bone loss by promoting osteogenic differentiation. Biomed. PharmacOther.

[bib0017] Guo J., Sun C., Qu L., Shen M., Dou T., Ma M., Wang K., Yang N. (2017). Genetic architecture of bone quality variation in layer chickens revealed by a genome-wide association study. Sci. Rep..

[bib0018] Habig C., Henning M., Baulain U., Jansen S., Scholz A.M., Weigend S. (2021). Keel bone damage in laying hens-its relation to bone mineral density, body growth rate and laying performance. Animals.

[bib0019] Harlander-Matauschek A., Rodenburg T.B., Sandilands V., Tobalske B.W., Toscano M.J. (2015). Causes of keel bone damage and their solutions in laying hens. World's Poult. Sci. J..

[bib0020] Hu A., Zhao X.T., Tu H., Xiao T., Fu T., Wang Y., Liu Y., Shi X.J., Luo J., Song B.L. (2018). PIP4K2A regulates intracellular cholesterol transport through modulating PI(4,5)P(2) homeostasis. J. Lipid Res..

[bib0021] Hu Y., Song F., Jiang H., Nuñez G., Smith D.E. (2018). SLC15A2 and SLC15A4 mediate the transport of bacterially derived di/tripeptides to enhance the nucleotide-binding oligomerization domain-dependent immune response in mouse bone marrow-derived macrophages. J. Immunol..

[bib0022] Hu Z.L., Park C.A., Wu X.L., Reecy J.M. (2013). Animal QTLdb: an improved database tool for livestock animal QTL/association data dissemination in the post-genome era. Nucleic Acids Res..

[bib0023] Huang d.W., Sherman B.T., Lempicki R.A. (2009). Systematic and integrative analysis of large gene lists using DAVID bioinformatics resources. Nat. Protoc..

[bib0024] Johnsson M., Wall H., Lopes Pinto F.A., Fleming R.H., McCormack H.A., Benavides-Reyes C., Dominguez-Gasca N., Sanchez-Rodriguez E., Dunn I.C., Rodriguez-Navarro A.B., Kindmark A., Koning D.J. (2022). Genetics of tibia bone properties of crossbred commercial laying hens in different housing systems. G3: Genes Genomes Genet..

[bib0025] Kim M.H., Song S.W., Kim K.S. (2019). Abdominal obesity is associated with lower bone mineral density in non-weight-bearing site in Korean men. Am. J. Mens Health.

[bib54] Kumar A., Sharma R.K., Singh H., Singh C.V., Singh B. (2002). Genetic studies on some economic traits of Rhode Island Red. Indian J. Poult. Sci..

[bib53] Li Y.D., Liu X., Li Z.W., Wang W.J., Li Y.M., Cao Z.P., Luan P., Xiao F., Gao H.H, Guo H.S., Wang N., Li H. (2021). A combination of genome-wide association study and selection signature analysis dissects the genetic architecture underlying bone traits in chickens. Animal.

[bib0026] Liu W., Li D., Liu J., Chen S., Qu L., Zheng J., Xu G., Yang N. (2011). A genome-wide SNP scan reveals novel loci for egg production and quality traits in white leghorn and brown-egg dwarf layers. PLoS One.

[bib0027] Lopez, D.M., 2018. Characterisation of the major facilitator superfamily domain containing 1 protein (MFSD1) and study of its physiological role in the mouse. Christian-Albrechts-Universität zu Kiel. urn:nbn:de:gbv:8-diss-242968.

[bib0028] Lv S., Zhang A., Di W., Sheng Y., Cheng P., Qi H., Liu J., Yu J., Ding G., Cai J., Lai B. (2016). Assessment of fat distribution and bone quality with trabecular bone score (TBS) in healthy Chinese men. Sci. Rep..

[bib0029] Massa Lopez D., Damme M., Saftig P. (2016). Characterising the role of the lysosomal membrane proteins MFSD1 and TMEM106b in osteoclasts. Bone Abstr..

[bib0030] Moore C.M., Jacobso S.A., Fingerlin T.E. (2019). Power and sample size calculations for genetic association studies in the presence of genetic model mis-specification. Hum. Hered..

[bib0031] Mueller W.J., Brubaker R.L., Caplan M.D. (1969). Egg shell formation and bone resorption in laying hens. Federation Proc..

[bib0032] Nassar M.K., Goraga Z.S., Brockmann G.A. (2012). Quantitative trait loci segregating in crosses between New Hampshire and White Leghorn chicken lines: II. Muscle weight and carcass composition. Anim. Genet..

[bib0033] Podisi B.K., Knott S.A., Dunn I.C., Burt D.W., Hocking P.M. (2012). Bone mineral density QTL at sexual maturity and end of lay. Br. Poult. Sci..

[bib0034] Purcell S., Neale B., Todd-Brown K., Thomas L., Ferreira M.A.R., Bender D., Maller J., Sklar P., de Bakker P.I.W., Daly M.J., Sham P.C. (2007). PLINK: a toolset for whole-genome association and population-based linkage analysis. Am. J. Hum. Genet..

[bib0035] Raymond B., Johansson A.M., McCormack H.A., Fleming R.H., Schmutz M., Dunn I.C., De Koning D.J. (2018). Genome-wide association study for bone strength in laying hens. J. Anim. Sci..

[bib0036] Refaey M.E., McGee-Lawrence M.E., Fulzele S., Kennedy E.J., Bollag W.B., Elsalanty M., Zhong Q., Ding K.H., Bendzunas N.G., Shi X.M. (2017). Kynurenine, a tryptophan metabolite that accumulates with age, induces bone loss. J. Bone Miner. Res..

[bib0037] Rosen C.J., Bouxsein M.L. (2006). Mechanisms of disease: is osteoporosis the obesity of bone?. Nat. Clin. Pract. Rheumatol..

[bib0038] Sallam M., Wilson P.W., Andersson B., Schmutz M., Benavides C., Dominguez Gasca N., Sanchez Rodriguez E., Rodriguez Navarro A.B., Dunn I.C., De Koning D.J., Johnsson M. (2023). Genetic markers associated with bone composition in Rhode Island Red laying hens. Genet. Sel. Evol..

[bib0039] Sallam M., Wall H., Wilson P.W., Andersson B., Schmutz M., Benavides C., Checa M., Sanchez‑Rodriguez E., Rodriguez‑Navarro A.B., Kindmark A., Dunn I.C., de Koning D.J., Johnsson M. (2025). Genomic prediction of bone strength in laying hens using different sources of information. Animal.

[bib0040] Schreiweis M.A., Hester P.Y., Moody D.E. (2005). Identification of quantitative trait loci associated with bone traits and body weight in an F2 resource population of chickens. Genet. Sel. Evol..

[bib0041] Sherman B.T., Hao M., Qiu J., Jiao X., Baseler M.W., Lane H.C., Imamichi T., Chang W. (2022). DAVID: a web server for functional enrichment analysis and functional annotation of gene lists (2021 update). Nucleic Acids Res..

[bib0042] Sun Y., Li J., Xie X., Gu F., Sui Z., Zhang K., Yu T. (2021). Macrophage-osteoclast associations: origin, polarization, and subgroups. Front. Immunol..

[bib0043] Teufel S., Hartmann C. (2019). Wnt-signaling in skeletal development. Curr. Top. Dev. Biol..

[bib0044] Turner S.D. (2018). qqman: an R package for visualizing GWAS results using Q-Q and manhattan plots. J. Open Source Softw..

[bib0045] Wang C.G., Liao Z., Xiao H., Liu H., Hu Y.H., Liao Q.D., Zhong D. (2019). LncRNA KCNQ1OT1 promoted BMP2 expression to regulate osteogenic differentiation by sponging miRNA-214. Exp. Mol. Pathol..

[bib0046] Wang J.Z., Zhao B.H. (2021). MiR-23b-3p functions as a positive factor for osteoporosis progression by targeting CCND1 in MC3T3-E1 cells. In Vitro Cell Dev. Biol. Anim..

[bib0047] Wang L.J., Cai H.Q. (2020). Let-7b downgrades CCND1 to repress osteogenic proliferation and differentiation of MC3T3-E1 cells: an implication in osteoporosis. Kaohsiung J. Med. Sci..

[bib0048] Webster A.B. (2004). Welfare implications of avian osteoporosis. Poult. Sci..

[bib0049] Wu M., Chen G., Li Y.P. (2016). TGF-β and BMP signaling in osteoblast, skeletal development, and bone formation, homeostasis and disease. Bone Res..

[bib0050] Yadav V.K., Ryu J.H., Suda N., Tanaka K.F., Gingrich J.A., Schütz G., Glorieux F.H., Chiang C.Y., Zajac J.D., Insogna K.L. (2008). Lrp5 controls bone formation by inhibiting serotonin synthesis in the duodenum. Cell.

[bib0051] Zhang K., Shi Z., Ren Y., Han X., Wang J., Hong W. (2021). Kcnq1ot1 promotes osteogenic differentiation and suppresses osteoclast differentiation. J. South Med. Univ..

[bib0052] Zhou X., Stephens M. (2012). Genome-wide efficient mixed-model analysis for association studies. Nat. Genet..

