# Supplementary figures and images for: Genetic markers associated with bone strength and density in Rhode Island Red laying hens
Source: Poult Sci. 2025 May 2;104(7):105246. doi: 10.1016/j.psj.2025.105246 (PMC12138422; doi:10.1016/j.psj.2025.105246)

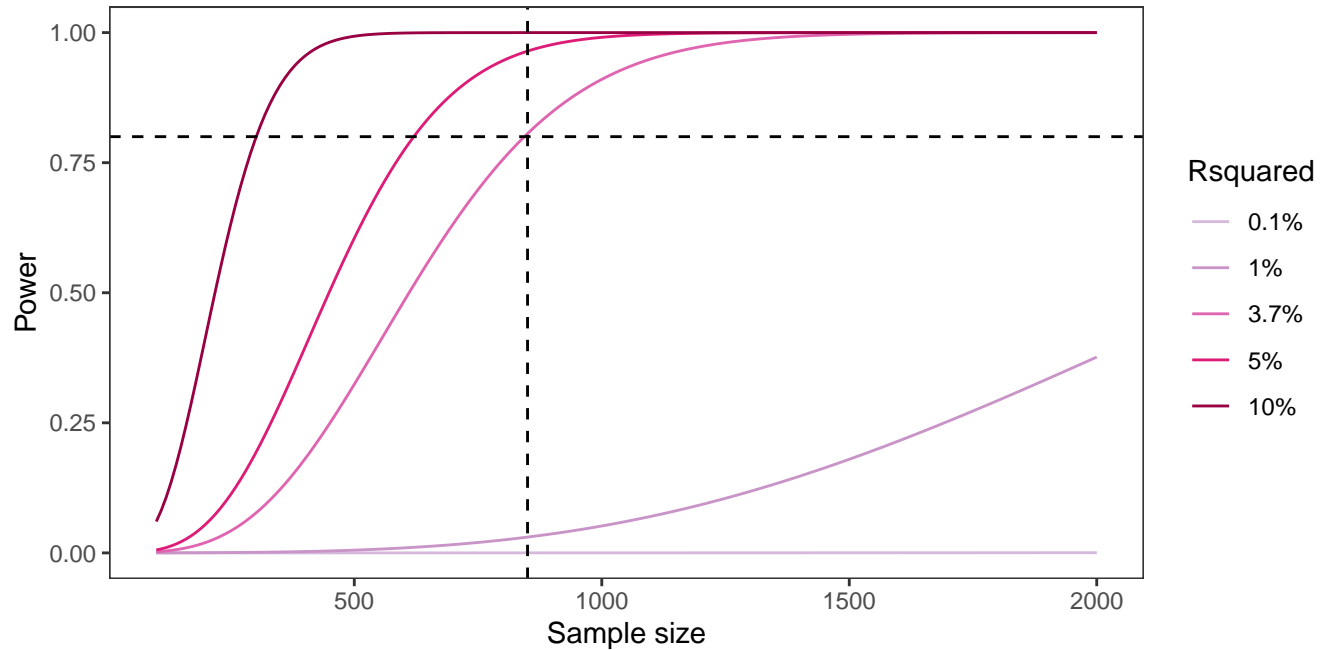

Supplement: Supplementary file 1 [file mmc1.pdf]
